# Emerging adults in substance misuse intervention: preintervention characteristics and responses to a motivation-enhancing program

**DOI:** 10.1186/s13722-016-0064-7

**Published:** 2016-11-09

**Authors:** Blair Beadnell, Michele A. Crisafulli, Pamela A. Stafford, Erin A. Casey

**Affiliations:** 1 Prevention Research Institute, 841 Corporate Drive, Suite 300, Lexington, KY 40503 USA; 2 University of Maryland, Baltimore County, 1000 Hilltop Circle, Baltimore, MD 21250 USA; 3 Social Work Program, University of Washington (Tacoma), 1900 Commerce Street, Box 358425, Tacoma, WA 98402 USA

**Keywords:** Prevention, Emerging adults, Latent transition analysis, Alcohol, Alcohol use disorder, Motivation-enhancing

## Abstract

**Background:**

Emerging adulthood is an age of particularly risky behavior. Substance misuse during this phase of life can be the beginning of longer-term problems, making intervention programs particularly important. This study’s purposes were to identify alcohol use profile subgroups, describe the preintervention characteristics of each, and assess how many participants transitioned to lower-risk profiles during the course of the intervention.

**Methods:**

We used latent transition analyses to categorize 1183 people court ordered to attend Prime For Life^®^ (PFL), a motivation-enhancing program, into preintervention and postintervention profiles. We then assessed how many made transitions between these profiles during the course of the intervention.

**Results:**

Profiles included two low-risk statuses (abstinence and light drinking) and two high-risk statuses (occasional heavy drinking and frequent heavy drinking). We found that people in profile subgroups that reflected heavier 90-day preintervention drinking were likely to transition to profiles reflecting postintervention intentions for lower-risk drinking in the subsequent 90 days. In contrast, the likelihood of transitioning from a lower-risk to a higher-risk profile was extremely low. These positive changes were found for people of both sexes and for those above versus below the legal drinking age, albeit for more women than men in the heaviest drinking group.

**Conclusions:**

Findings showed positive changes during intervention for many emerging adult participants attending PFL. Further research is needed that include comparison conditions, as well as examine longer-term outcomes in this population.

## Background

Emerging adulthood (EA), a developmental period situated between adolescence and adulthood, is a time of particular concern with substance use. Initially conceptualized by Arnett in 2000, EA is typically seen as lasting from approximately age 18–25 (though in some cases through the late twenties) [1]. The characteristics of this developmental stage include being a period of identity exploration, instability, self-focus, transition, and optimism. These elements contribute to it being a time of high risk-taking behaviors [2]. In particular, the early twenties is a period during which rates of alcohol use and related problems increase to their highest point [3–5].

### Classification of drinking patterns among emerging adults

Researchers have added to knowledge on alcohol use by using methods that take into account that drinking behavior is best reflected not by any one aspect, but by a combination. While alcohol research typically examines both quantity (the amount of alcohol consumed) and frequency (how often alcohol is used), these are often examined separately. However, looking at these together to see drinking patterns can more accurately determine individuals’ level of risk. Accordingly, classification techniques such as cluster analysis and mixture modeling (e.g., latent class and latent transition analysis) help investigators identify subgroups with unique alcohol use profiles based on typical quantity and frequency.

In the EA population, fairly consistent subgroups representing gradations of risky drinking emerge when using these techniques. There is a common thread across studies of finding lower-risk profiles (no use and occasional low use groups) and higher-risk profiles (occasional high use and frequent high use groups). For example, Auerbach and Collins identified five groups that showed varying levels of risk among individuals 18.5–22.5 years old: no use, occasional low use, occasional high use, frequent high use, and frequent high use with heavy episodic drinking [6]. Cleveland and colleagues found four drinking patterns among college students (i.e., nondrinkers, weekend nonbingers, weekend bingers, and heavy drinkers) and among non-college 18–22 year olds (i.e., nondrinkers, weekend light drinkers, weekend risky drinkers, and daily drinkers) [7, 8]. These profiles showed predictive validity in that those with the heavier drinking profiles were more likely to show negative consequences from drinking (e.g., feeling sick, missing work, driving while impaired, and getting into fights) [8].

Using these classification techniques, sex and age emerge as important factors related to EA drinking patterns and responsiveness to intervention. In one study using growth mixture modeling to categorize intervention efficacy, older and female college students were more likely to respond favorably [9]. Similar patterns appear in research of naturally-occurring drinking trajectories (i.e., outside of the intervention context). One study found that EA women were underrepresented in categories showing continued high use or increased use with age, while overrepresented in a category of never having problematic use [10]. In terms of age, questions remain about the trajectories along which drinking evolves over the years of EA. There is evidence that a heavier drinking pattern when below the legal drinking age predicts continuation in such a pattern once the legal drinking age is reached [6]. As such, problematic drinking at younger ages may be an indicator of future problems. However, while partially predictive, drinking early in EA does not necessarily determine an individual’s later status. For example, Schulenberg and Magos [10] reviewed studies showing changes in drinking trajectories can occur such that earlier heavy drinking patterns can resolve later in people’s developmental trajectory. Given these indications that sex and age might play roles in EA drinking trajectories, their influence is an important topic to investigate.

Classification techniques can be useful for informing applied intervention research in two ways. One is by allowing for examination of participants’ preintervention substance use patterns, as opposed to relying on individual variables. This more nuanced information about groups of participants presenting for intervention may help program design and implementation. For example, optimally effective intervention content might differ for target groups whose high-risk use is regular versus episodic, or when both patterns exist. Another way classification techniques are useful is by providing information on the clinical significance of changes made by participants. Specifically, longitudinal classification methods like latent transition analysis (LTA) allow calculation of the percentage of people making meaningful changes. These sorts of analyses are a useful supplement to traditional tests that focus on the statistical significance of average changes over time and, moreover, respond to calls for examination of the practical utility of intervention programs [11, 12]. This study capitalized on the usefulness of classification methods by examining initial changes during a motivation-enhancing (ME) intervention among EAs receiving an indicated substance use prevention program.

### Motivation enhancing substance use interventions and emerging adults

Intervention methods often examined with EAs engaging in high-risk behaviors include cognitive-behavioral approaches, particularly those incorporating methods targeting motivation for change. Specifically, many indicated prevention programs incorporate principles from motivational interviewing (MI) and are thus often called motivation-enhancing (ME) approaches. Both ME and MI approaches involve a collaborative counseling style intended to strengthen commitment to change, particularly by eliciting and building on participants’ already existing motivation for change [13].

Theoretically, ME interventions should be especially well-suited for EAs because they provide a framework for working with the resistance to authority and ambivalence about change that often characterize this developmental period [2]. Indeed, research has shown preliminary support for ME with EAs. Specifically, several studies have shown that interventions incorporating ME elements can decrease substance use and/or consequences among college students [14–17]. Additionally, a recent meta-analysis showed that brief ME and cognitive-behavioral interventions are both associated with improvements in alcohol consumption and consequences among young adults, although effects were attenuated to non-significance by 2-year follow-up [18].

The Transtheoretical Model (TTM) [19] provides a theoretical lens through which to view the meaning and importance of immediate gains achieved during the course of ME interventions such as the one examined in this study. The model posits that intentional behavior change involves moving through multiple stages and completing numerous cognitive and behavioral tasks along the way. ME interventions are often geared toward individuals in earlier stages of change, particularly precontemplation (where individuals have no intention of making changes) and contemplation (where they are considering but ambivalent about making changes). In such cases, a successful short-term outcome would be movement to a later stage of change, such as preparation, in which people have made a decision to change and begun preparing to do so (but have not yet necessarily made any behavioral changes). The model sees such movement as an important step in setting the stage for behavioral change. However, this does not guarantee longer-term success: meta-analyses show only moderate correlations between intentions and behavior ranging from .40 to .82 (overall *r* = .53) [20]. Nevertheless, intentions have meaning in that the TTM and other influential health behavior theories (e.g., the Theory of Reasoned Action and the Theory of Planned Behavior) see them as necessary precursors to subsequent behavior change [21, 22].

### Study purpose

While ME programs are theoretically well-suited to and have shown promise with this age group, little is known about changes EAs make when participating in these approaches. This includes understanding what drinking profile subgroups exist in an intervention population, in what proportions, and whether some are more versus less likely to show improvements during the course of the ME intervention. Additionally, while potentially important given the previously mentioned findings about age and sex, little is known about whether demographic characteristics are related to preintervention profiles, and changes made during intervention.

To examine these questions, this study focused on initial change from baseline to immediately postintervention among EAs in an ME substance use intervention. We analyzed data from individuals attending Prime For Life^®^ (PFL), a group-delivered ME indicated prevention program. Participants had all experienced negative consequences of substance use in the form of being arrested for driving while intoxicated, and all were required to complete the program to regain driving privileges. Described in more detail below, PFL focuses on encouraging the reduction of negative social, legal, occupational, and health consequences from alcohol and drug misuse. A previous data collection showed PFL to be effective in producing short-term changes in cognitive outcomes [23]. Another study showed that such changes are meaningful and translate into longer-term improvements: in an examination of driving records of PFL attendees in a 2-day version of the program, PFL participants had lower 3-year recidivism rates compared to standard care conditions [24]. In both datasets, changes occurred for emerging adults as well as the broader range of adult participants. Moreover, moderation analyses showed that improvements in cognitive outcomes (e.g., understanding tolerance, perceived risk for addiction, perceived risk for negative consequences, and problem recognition) were similar or greater for EA participants. In terms of recidivism, moderation analyses showed that PFL’s lower rearrest rates occurred for 18–29 year olds in the same way as for other adults receiving PFL, although this advantage of PFL over standard care did not extend to 18–29 year olds required to receive additional substance use treatment beyond the PFL.

The present study had three specific purposes. One was to identify subgroups based on patterns of preintervention alcohol use in a sample of EAs attending a court-ordered intervention (PFL). Second, we sought to quantify the extent to which participants transitioned from previous patterns of heavy alcohol use to postintervention patterns characterized by intentions to use substances less in the future. Third, we wished to determine whether sex and being of legal drinking age were predictors of these transitions to lower alcohol use intentions.

We guided our interpretation of results based on drinking guidelines taught in PFL. Known as the 0–1–2–3 guidelines, these define the amount of drinking considered “low risk.” The low-risk guidelines for alcohol were the same for both women and men, and included no more than one standard drink (defined as ½ ounce of pure alcohol at the time of this data collection) in an hour, two standard drinks daily, or three standard drinks on any day. Based on this, we considered drinking less than three drinks in a day as low risk, and four or more as high risk. Although the guidelines have additional aspects (e.g., a peak amount per week as 14 standard drinks, and abstinence for those who have already developed an alcohol use disorder) we did not have data to consider these.

## Methods

### Participants

Participants (*N* = 1183) came from ten states in the U.S. (Georgia, Iowa, Indiana, Kentucky, North Carolina, Montana, North Dakota, South Carolina, Tennessee, and Utah), and had been arrested for impaired driving. All were required to attend the program (either automatically or from being court-ordered) and completed it. These states use PFL for arrestees, with program completion required as a prerequisite for reinstating driving privileges. Participants received PFL from impaired driving schools or substance abuse treatment agencies in 2011 and 2012. Participants paid the program cost out of pocket, and the price varied depending on the state (states typically mandate the maximum cost agencies can charge for program attendance). Cost typically ranged from none (if public funding was available to a participant), or anywhere between $150 and $500.

### Procedure

Using structured protocols and scripts, PFL instructors distributed paper and pencil questionnaires to the group at the beginning of the first session and immediately after the last session ended. Data collection methods prevented instructors from seeing participant responses. The length of time between administration of baseline and posttest questionnaires varied due to state regulations from 2 to 14 days (with the intervention being 12–20 h).

### Materials and measures

The previously pilot tested questionnaires took approximately 15 min to complete. Questions asked about number of drinks in a 90 day period and provided responses ranging from “0” to “24 or more,” except where otherwise noted. Questionnaires defined one standard drink following the PFL risk guidelines used at the time (12 oz of beer, 4 oz of wine, or 1.25 oz of 80-proof alcohol). In a previous psychometric evaluation, test retest reliability of items ranged from *r* = .72–.94.

#### Demographics

Participants reported on their sex and age. For analyses, we dichotomized age as being legal drinking age (21–25) or not (18–20).

#### Alcohol consumption and intentions

Items were drawn from epidemiological studies [25, 26] about the quantity and frequency of drinking. Questions regarding preintervention drinking asked about the 90 days of behavior prior to attending PFL; at posttest, the same questions were asked but about intentions for the next 90 days.

The preintervention quantity items were “In the 90 days before this program, on days when I drank, I usually had…”. Participants could choose any number from 0 to 24 or more, and for analyses we created an ordinal variable of 0, 1–3, 4–6, and 7 or more. This categorization captured variation in drinking in a parsimonious way while aligning to the PFL drinking guidelines taught in the program (e.g., with the 0 and the 1–3 categories falling within what is considered low risk; and the other categories representing two levels of higher-risk drinking). Frequency items assessed frequency of drinking above the PFL guidelines using the introductory statement “In the 90 days before this program” and two items: “I drank 4–6 drinks” and “I drank 7 or more drinks.” Response categories were “not at all,” “less than once a week,” “about once a week,” “2–3 days a week,” “4–6 days a week,” and “most days.” We categorized these as “never,” “less than once a week,” and “one or more times a week.” Questionnaires used the same item wording and response option coding for postintervention future intentions by changing the introductory statements (e.g., “In the next 90 days, I intend to …”).

For alcohol use during the 90-day period before intervention, we used postintervention retrospective responses about drinking that occurred during those 90 days before PFL. We based this decision on previous research indicating that individuals experiencing legal issues based on their substance use and participating in court-ordered intervention report higher levels of previous use when asked following program participation than when queried about the same time period prior to the program [27, 28]. While we cannot be certain these postintervention-collected data were more accurate, we see reason to believe that it might be. Specifically, this population is subject to factors hypothesized to decrease the accuracy of self-report such as unhappiness about attending a court-ordered program, social desirability, and concerns about confidentiality given their legal entanglements [29]. We reasoned that these factors were likely to be less salient at the postintervention timepoint after participants became familiar with the instructors and program, which may have allowed for more accurate reporting.

### Description of Prime For Life

PFL is a theory-based, manualized, structured, and motivation-enhancing indicated prevention program. It provides information drawn from scientific research about hazards attendant to high-risk levels of substance use focusing on the importance of personal choice in preventing future problems. Negative consequences include health and impairment problems secondary to high-risk substance use and reflect all aspects of life. Specifically, participants identify the areas of life they value and then self-assess the extent to which their substance use threatens them. PFL does not solely promote abstinence but instead provides guidelines for low-risk use based on an extensive review of the literature examining health and impairment consequences [30]. The program helps participants assess their risk for substance dependence and develop individualized plans to change behavior.

PFL is an ME-based program, and therefore emphasizes the manner in which the program is delivered. It uses three specific empirically supported practices: (1) establishing an effective partnership between instructors and participants, (2) diffusing discord, and (3) providing clear direction [13]. The progress and activities of PFL are guided by the Lifestyle Risk Reduction Model [31], the Transtheoretical Model [32], and persuasion theory [33]. PFL is typically delivered in a group format, and program length varies from 12 to 20 h depending on requirements mandated in each state. Instructors always administer the same core program components, with only the number of optional activities varying. PFL instructors complete a new instructor training conducted by PRI staff and then a self-review process after their first group; most are licensed substance abuse professionals.

### Analysis strategy

We first conducted preliminary analyses (described later) to assess whether statistically significant changes occurred in which participants intended to drink less in the future than they had drank before attending the intervention. We then conducted LTA analyses in Mplus v7 guided by methods described by Collins and Lanza [34]. LTA is a statistical method for identifying subgroups, called statuses, of individuals at more than one timepoint. In addition, it estimates transition probabilities, which are the probabilities of transitioning from each Time 1 status to each Time 2 status. We treated preintervention drinking behavior and postintervention drinking intentions as two timepoints; referred here as baseline and posttest, respectively. We treated drinking variables as ordinal in the LTA and—to maximize interpretability of the results—fixed item-response probabilities to be equal across timepoints. Additionally, we specified intervention group membership as a clustering variable in multilevel aggregated analysis in order to adjust model parameters to correctly calculate standard errors [35].

As a first step, we compared models with varying numbers of statuses. We based our decisions about the optimal number of statuses by seeking a balance between statistical criteria (i.e., BIC, AIC, and entropy), parsimony, and theoretical interpretability and meaningfulness. As recommended by Collins and Lanza [34], we paid less attention to the *p* value for *G* ^2^ given that its distribution is not well represented by the Chi square distribution. In terms of other statistical criteria, the parametric bootstrapped likelihood ratio test is an often-used statistic that helps identify the optimal number of statuses. However, we were unable to use it because the test is not available in Mplus when using multilevel data or when there is more than one latent status variable. Thus, we instead chose to focus on the criteria described above. As a next step, we added sex and being of legal drinking age as predictors of baseline status and transition probabilities.

## Results

### Sample characteristics

Of the 4645 individuals who completed baseline questionnaires, 1199 met our inclusion criteria of being between 18 and 25 years old, having been arrested for impaired driving (rather than another substance use infraction such as drug possession), and having completed the PFL program. We included 1183 (98.7%) in all analyses after removing 16 with missing data (2 missing sex, 15 missing data on all drinking variables).

Most participants were male (69%) and Caucasian (81%). In terms of race/ethnicity, the rest identified as Asian (1%), Black (8%), Hispanic (5%), Native American (2%), Multiracial (3%), or “Other” (<1%). Participants ranged from 18 to 25 years old (*M* = 22, *SD* = 1.99), with 76% at or above the legal drinking age (21 or older). About three-quarters had never been married (76%). Educationally, 8% had not finished high school, 34% had completed high school or obtained a GED, 41% had attended but not completed college, 7% had a 2-year college degree, and 10% had a 4-year college or graduate degree. Of six items reflecting substance dependence symptoms experienced in the previous year (e.g., failing to meet normal expectations, drinking in the morning, trying but failing to cut down), 27% endorsed none, 39% one or two, and 34% three to six. Unfortunately, we do not have specific information on the amount of time between arrest and program enrollment; however, unpublished data from a separate PFL program evaluation suggest this varies widely (i.e., anywhere from less than 3 months to 2 years or more, with the majority being between 3 and 12 months).

### Missing data

Missing data rates on variables used as indicators of status membership were low among the analysis sample of 1183 and appeared to be due to occasional skipping of items. The amount missing on any one variable ranged from 0.8 to 2.1%, with no discernable pattern. Mplus uses maximum likelihood estimation, which allowed the inclusion of participants missing some variables.

### Preliminary analysis: comparison of preintervention drinking to future intentions at postintervention

Table 1 shows distributions of the drinking variables used in the LTA and comparisons of preintervention drinking to intended future drinking. Generalized Estimating Equation comparisons showed that participants intended to drink less in the future than they had in the past on each variable. In particular, many more participants reported intending to completely abstain or to drink in lower-risk amounts (1–3 drinks) than they had in the 90 days before attending the intervention.

**Table 1:** Distributions of drinking variables used in the latent transition analysis (*N* = 1183)

Alcohol use	Preintervention drinking (previous 90 days)		Intentions at postintervention (for next 90 days)		Intentions at postintervention compared to preintervention drinking^a^	
	%	*n*	%	*n*	*χ* ^2^ (*df* = 1)	*p*
Usual daily quantity					533.60	<.001
0 drinks	13.6	159	27.3	318		
1–3	22.3	261	50.3	586		
4–6	32.8	385	15.8	184		
7–9	14.0	164	3.8	44		
≥10	17.3	204	2.8	32		
Frequency 4–6					448.34	<.001
Never	29.7	348	60.7	712		
<Once a week	33.6	393	26.4	310		
≥Once a week	36.7	430	12.9	151		
Frequency ≥7					389.30	<.001
Never	43.5	504	73.9	862		
<Once a week	30.2	350	17.9	209		
≥Once a week	26.3	304	8.2	95		

^a^Computed using Generalized Estimating Equation (GEE) analysis

### Identification of latent statuses

Preliminary latent class analysis models showed the smallest BIC values for the three and four status models (7439.9 and 7436.4 for preintervention drinking, and 5577.3 and 5580.2 for intentions at postintervention, respectively) compared to the two and five status models at the same timepoints (7740.5 and 7466.2 for preintervention drinking, and 5746.6 and 5628.1 for intentions at postintervention, respectively). Entropy scores were acceptably high (>.80) regardless of the number of status groups. We found at both timepoints that the four status model provided the best theoretical interpretability and was the most informative.

We then proceeded to the LTA. Table 2 shows fit statistics for two through five status models. Here, the four and five status models had the lowest BIC values. Entropy values were uniformly high across models. While the five status model had the lowest AIC, we found that the additional status group did not add theoretical value and, thus, we favored the more parsimonious four status model.

**Table 2:** Model fit statistics for two to five status LTA models

Number of latent statuses	−*LL*	*G* ^2^	*df*	*AIC*	*BIC*	*Entropy*	Status counts^a^	
							Preintervention drinking	Intentions at postintervention
Two	6627.1	1945.2	1985	13,292.2	13,388.7	0.89	813, 370	349, 834
Three	6239.0	1245.3	1971	12,541.9	12,704.4	0.88	302, 502, 379	708, 138, 337
Four	6102.6	958.3	1955	12,299.1	12,537.7	0.86	365, 226, 135, 457	106, 388, 443, 246
Five	6041.7	854.1	1940	12,211.4	12,536.3	0.85	235, 136, 302, 225, 285	114, 452, 180, 383, 54

^a^Status counts based on most likely group membership

**Fig. 1:**
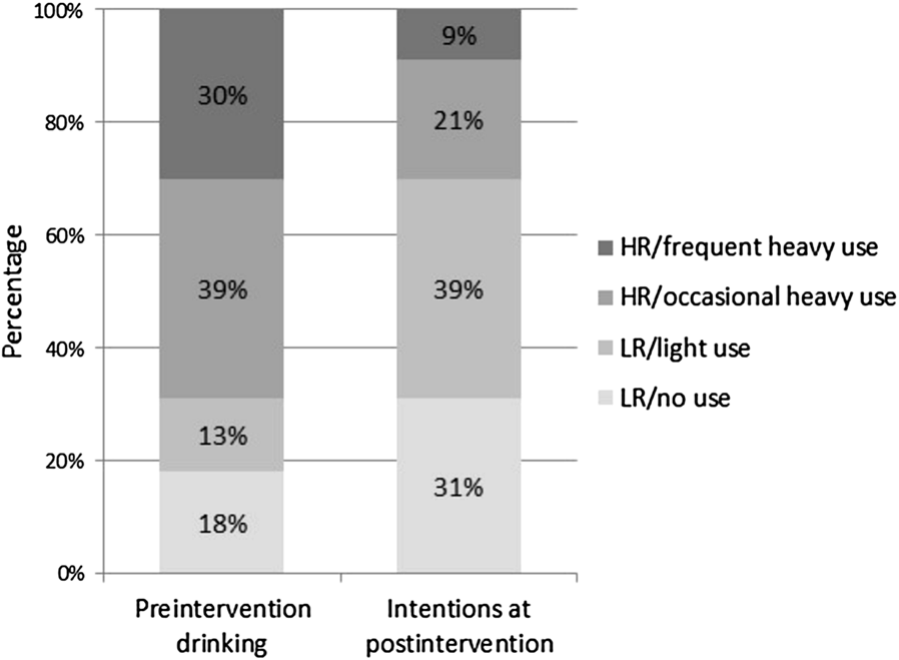
Percentage in their most likely LTA statuses based on preintervention drinking and future intentions at postintervention

Table 3 provides each status group’s drinking profile. The top of Table 3 summarizes the proportion of the sample belonging to each of the four status groups for both preintervention drinking and postintervention intentions, and Fig. 1 depicts this visually. As indicated earlier, preintervention profiles represented behavior in the 90 days before intervention, and postintervention profiles represented drinking intentions for the 90 days following PFL attendance. The table orders the profiles from least drinking to greatest drinking, and we labeled the groups to indicate low versus high risk (according to the guidelines taught in the PFL program). We labeled each status group according to what the item-response probabilities suggested were its preponderant characteristics. Two groups corresponded to low-risk (LR) drinking according to the PFL guidelines. The LR/no use group represented abstinence from drinking. The LR/light use group profile involved drinking, with the usual number of drinks being 1–3, relatively low probability of drinking 4–6 drinks, and no probability of drinking 7 or more drinks. The other two groups showed high risk (HR) by the usual number of drinks and frequency of binge drinking falling outside the PFL guidelines. The HR/occasional heavy use group was characterized by some probability of having a usual number of drinks within the guidelines (1–3) but a higher probability of being outside of them (4–6). Heavy drinking (4–6, and 7 or more drinks) was likely to occur fairly infrequently (less than once a week). The remaining profile, the HR/frequent heavy use status, was one with a high probability of a usual number of drinks outside the guidelines and this occurring at least once a week.

**Table 3:** Characteristics of status groups from LTA model (*N* = 1183)

Variables	Status group			
	Low risk (LR)		High risk (HR)	
	No use	Light use	Occasional heavy use	Frequent heavy use
*Proportion in each status*				
Preintervention drinking (previous 90 days)	0.18	0.13	0.39	0.30
Intentions at postintervention (for next 90 days)	0.31	0.39	0.21	0.09
*Item-response probabilities* ^*a*^				
Usual quantity^b^				
0	0.77	0.00	0.02	0.02
1–3	0.11	0.97	0.29	0.08
4–6	0.09	0.03	0.53	0.26
7–9	0.02	0.00	0.11	0.24
≥10	0.01	0.00	0.05	0.40
Frequency 4–6^c^				
Never	1.00	0.73	0.03	0.10
<Once a week	0.00	0.27	0.70	0.11
≥Once a week	0.00	0.00	0.27	0.79
Frequency ≥7^c^				
Never	1.00	1.00	0.30	0.03
<Once a week	0.00	0.00	0.70	0.13
≥Once a week	0.00	0.00	0.00	0.84

^a^Item-response probabilities can be thought of as the frequency distribution for each variable within that status. These were the same for both preintervention drinking and postintervention intentions (they were constrained to be equal across timepoints). We labeled each status group according to the preponderant characteristics suggested by the item-response probabilities

^b^Prior/intended usual quantity of drinks

^c^Prior/intended frequency of consuming 4–6 or ≥7 drinks

Table 4 shows the transition probabilities. In other words, each row in Table 4 shows the likelihood that people in a particular preintervention drinking status either remained there or transitioned to a different status regarding their intentions at postintervention. People in both LR status groups typically remained in the same status postintervention. Specifically, 93 and 84% of LR/no use and LR/light use individuals, respectively, remained in the same status. Of the remaining LR/light use participants, 15% transitioned to the LR/no use status. In terms of people in HR groups, many transitioned to lower-risk statuses. For example, those in the HR/occasional heavy use status at baseline generally transitioned to the LR/no use and LR/light use statuses (22 and 47%), but infrequently to the HR/frequent heavy use status (1%). There was no single most common transition made by people in the HR/frequent heavy use status: 26% remained and another 32% transitioned to the other HR status. In other words, 58% of people in the highest-risk status at baseline continued to exhibit high-risk intentions postintervention, although they did most often show improvement to intending occasional heavy use rather than to maintain their frequent heavy use. The remaining 20 and 22% transitioned to the LR statuses.

**Table 4:** Transition probabilities from preintervention drinking to future intentions at postintervention (*N* = 1183)

Preintervention drinking status (previous 90 days)	Intentions at postintervention status (for next 90 days)			
	Low risk (LR)		High risk (HR)	
	No use	Light use	Occasional heavy use	Frequent heavy use
LR/no use	0.93	0.03	0.02	0.02
LR/light use	0.15	0.84	0.00	0.01
HR/occasional heavy use	0.22	0.47	0.30	0.01
HR/frequent heavy use	0.20	0.22	0.32	0.26

### Prediction by sex and legal drinking age

**Fig. 2:**
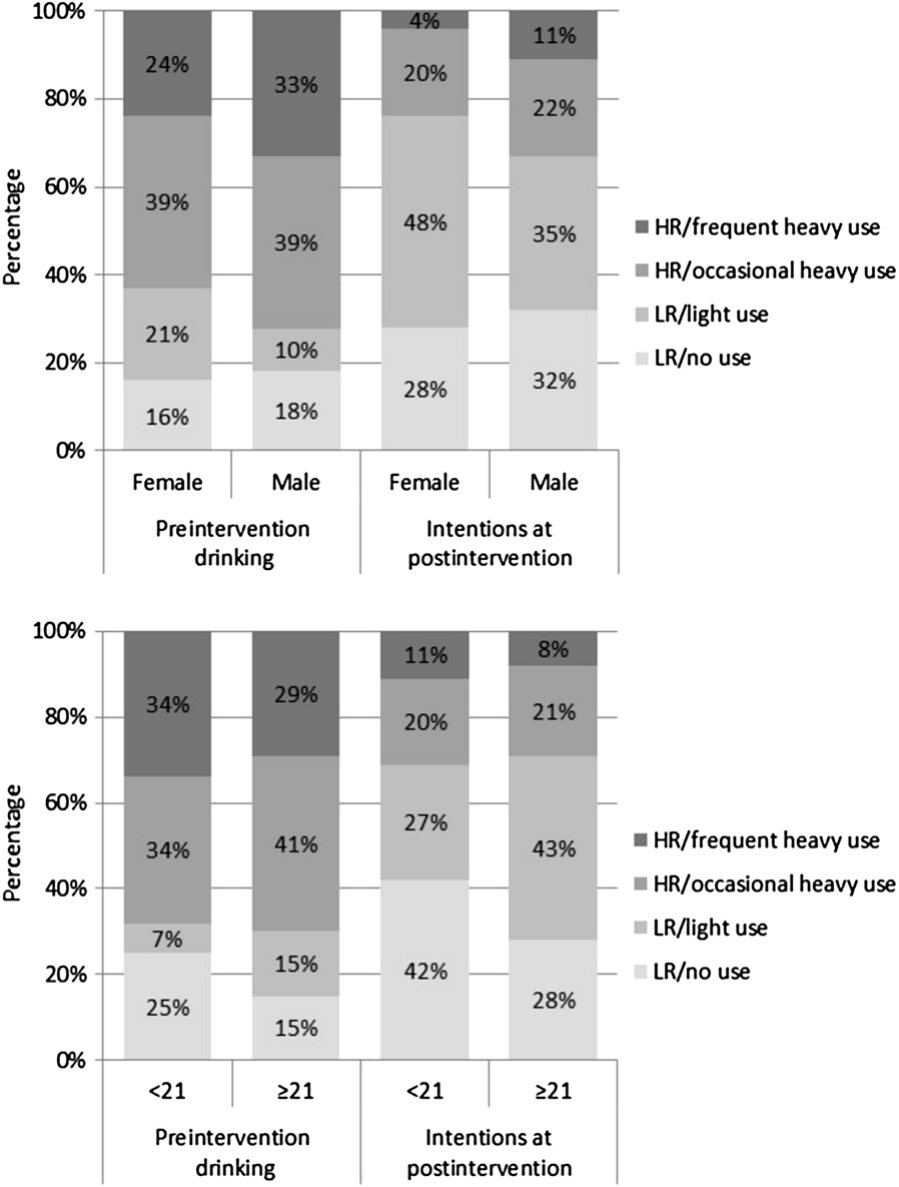
Percentage in their most likely LTA statuses based on preintervention drinking and future intentions at postintervention, by sex and age

As mentioned, sex and being of legal drinking age were included in the LTA as predictors of preintervention drinking status and transitions to statuses representing future intentions at postintervention. In multinomial logistic regression, sex was not related to preintervention status group membership. Specifically, females were not statistically significantly more likely to be in the preintervention LR/light use, HR/occasional heavy use, or HR/frequent heavy use statuses than they were to be in LR/no risk status; *odds ratios* (*OR*) = 1.69, 1.01, and 0.71, *p* = .07, .97, and .11; respectively. Table 5 shows the prediction of transition probabilities to relatively lower-risk statuses. Being female predicted greater likelihood of improvement in terms of transitioning from the HR/frequent heavy use status to each remaining status. For example, the table shows that women were more likely than men to transition from the HR/frequent heavy use to the LR/no use group (*OR* = 3.02). Otherwise, females and males had similar probabilities of improving. Figure 2 shows the most likely status memberships broken out by sex. This illustrates the net effects of sex on preintervention drinking status and subsequent transitions. As can be seen, while a relatively small number of participants remained in the HR/frequent heavy use status, women were particularly unlikely to do so.

**Table 5:** Odds ratios for sex and age predicting the transitioning to each lower risk status

Preintervention drinking status (previous 90 days)	Intentions at postintervention status (for next 90 days)			
	Low risk (LR)		High risk (HR)	
	No use	Light use	Occasional heavy use	Frequent heavy use
Effect of being female, compared to male				
LR/no use	–	–	–	–
LR/light use	2.65	Reference^a^	–	–
HR/occasional heavy use	0.73	1.56	Reference^a^	–
HR/frequent heavy use	3.02**	3.29**	2.78*	Reference^a^
Effect of being legal drinking age, compared to <21 years				
LR/no use	–	–	–	–
LR/light use	2.56	Reference^a^	–	–
HR/occasional heavy use	0.54	1.79	Reference^a^	–
HR/frequent heavy use	1.27	1.47	1.91	Reference^a^

* *p* < .05; ** *p* < .01

^a^Remaining in the same status group was the reference condition for each odds ratio. Said differently, each odds ratio represents the odds of switching to the lower-risk status group versus staying in the original status group

Those of legal drinking age were more likely to be in the preintervention LR/light use and HR/occasional heavy use statuses compared to the LR/no use status; *OR* = 3.55 and 1.90, both *p* < .01. However, age was not associated with greater likelihood of membership in the HR/frequent heavy use status; *odds ratio* = 1.30, *p* = .22. As shown in Table 5, being of legal drinking age did not statistically significantly predict subsequent transitions. Figure 2 shows the net effects of age on preintervention status and subsequent transitions. Most notably, people above and below the drinking age were equally likely to be in one of the LR postintervention intentions statuses. However, those over the legal drinking age were more likely to be in the LR/light use rather than the LR/no use status.

## Discussion

This study focused on the substance use patterns of emerging adults, a group particularly likely to use substances in high-risk and detrimental ways. We categorized participants with impaired driving offenses into substance use profiles reflecting their drinking in the 90 days before the start of their attendance at an indicated prevention program. We then showed transitions between these profile groups and others that were based on drinking intentions reported at postintervention for the following 90 days. Additionally, we assessed whether initial status memberships and transitions differed by sex and age group. The overall finding was that many participants attending PFL, a motivation-enhancing (ME) program, showed transitions to lower-risk profiles. This was true for people of both sexes and age groups, albeit with some variation for the heaviest drinking males compared to females.

In light of the challenges in effectively intervening with emerging adults, these are encouraging findings in terms of people during this developmental stage showing movement to increased readiness to change. The use of LTA in this study allowed us to provide more nuanced and clinically meaningful information than is sometimes provided in traditional null hypothesis testing. Specifically, it deepens understanding of the characteristics of EAs that interventionists can expect in such contexts, who among them makes changes and in what ways, and the role demographics may play in influencing these changes in this important population.

Knowledge of drinking profiles among EAs attending interventions is of practical relevance to interventionists. For instance, it is important to understand that, even prior to intervention, EA participants exhibit drinking patterns reflecting a range of severity from abstinence to frequent heavy use. In light of prior literature on EA alcohol use, it is not surprising that occasional and frequent heavy drinkers comprised the largest percentage of the sample. Nonetheless, almost a third reported lower risk either in the form of abstinence or, at most, light drinking. It may seem odd at first that such individuals exist in a sample arrested for impaired driving, and this may come as a surprise to interventionists used to working with EAs. One explanation might be that, for some, the impaired driving was due to illicit drug use rather than drinking. However, that is an unlikely explanation given that law enforcement has not yet perfected methods for detecting drug use among drivers. More likely is that the arrest, or its legal and social consequences, provided a catalyst for some that led to behavior change, whether achieved alone or after seeking outside assistance. For individuals attending the intervention 90 days or more after their arrest, such behavior change would have already occurred before the period assessed in this study’s baseline measurement and thus previous higher-risk use would not be captured.

As mentioned earlier, the Transtheoretical Model [19] provides a useful framework for understanding the implications of these findings for interventions with EAs. Postintervention statuses were based on future intentions and thus provide an indicator of readiness to change, including participant goals for that change. As such, this is conceptually consistent with the stages of change. Knowing that these status transitions occur may offer practical assistance to professionals who design or provide substance use intervention programs. Specifically, this information can guide the choices made about which intervention elements are needed, and in what circumstances, during the course of working with EAs. For example, the transition from HR/frequent use to HR/occasional use reflects an intention to change, but not an intention to move to low-risk use. In this case, there would be three primary practitioner tasks: (1) express and explore concerns about continued high-risk use while supporting client autonomy; (2) help develop plans for achieving the client’s desired change in frequency of high-risk use; and (3) help create a method for the client to evaluate the plan’s effectiveness in achieving long-term goals. Conversely, when clients remain in LR/no use—essentially an action or maintenance stage of change—the practitioner’s task is to help them identify potential threats to their action plan and revise as needed. The difference between these two examples emphasizes the need for interventionists to avoid assuming their audience is composed largely of people actively drinking in high-risk amounts. Rather, it is important to be aware of and address the broad range of people receiving services. As a result, group-based interventions should contain content that both supports those who have already begun making changes and increases motivation for change among those who have not [36]. As always, interventionists providing individual one-on-one interventions should carefully assess current drinking patterns and match counseling goals to the appropriate stage of change [32].

Results indicate that many individuals have lower-risk intentions for their future drinking relative to their preintervention alcohol use behavior. While over two-thirds reported either occasionally or frequently drinking in high-risk amounts immediately prior to PFL, less than a third intended to do so in the 90 days immediately following intervention. Moreover, we see two specific, positive patterns in terms of the actual transitions from each preintervention drinking to postintervention intentions status. First, those in preintervention LR statuses were likely to remain in the corresponding postintervention status, with transition to a higher-risk drinking profile being extremely rare. Second, many in HR statuses transitioned to a status reflecting future intentions of lesser drinking. For example, only a quarter of participants in the worst-case profile (the HR/frequent heavy use status) remained there. The rest transitioned to a lower-risk intention status, albeit with some only moving so far as the other HR intention status (i.e., intending occasional as opposed to frequent heavy use). While a change to intending less frequent high-risk use does not obviate potential hazards, it does reduce the overall risk and reflects an important public health change when spread over a population of heavy users, who experience a disproportionate number of consequences secondary to their high-risk use.

One interpretation of these transitions is that they reflect people’s preferred strategy for decreasing their risk. If so, the results suggest continuing to drink, but in lower-risk amounts, is a somewhat more popular choice for women, those of legal drinking age, and people whose previous high-risk drinking was only occasional. In contrast, men were likely to intend to lower their risk through abstinence as commonly as through light use. Similarly, those under the legal drinking age were more likely to lean towards abstinence rather than light drinking as a way of lowering risk. Interestingly, it appears that people who are frequent heavy drinkers preintervention express a range of reduction goals at postintervention. Some may prefer to occasionally drink heavily in the future while others may prefer to drink in lighter amounts or to be abstinent. Future research might explore such choices more thoroughly, including looking at what characterizes people preferring these different risk reduction strategies and how successful they are at achieving their stated goals.

A small number of participants who were in HR statuses at preintervention—both occasional and frequent heavy drinkers—remained in the corresponding status in terms of their postintervention intentions. Although we cannot know whether this study’s observed transitions were caused by the intervention (since there was no comparison condition), to the extent that they were these participants appear to have responded less well. This is a reminder that decreasing alcohol-related risk may be particularly challenging among EAs, given they are in what is typically the heaviest drinking period of life. While we did not find that being of legal drinking age was associated with transition from HR statuses, women were more likely than men to transition from the HR/frequent heavy use to lower-risk statuses. Although many EA men with this preintervention drinking pattern did transition from this status, our finding suggests there may be a subgroup among them that is particularly challenging to influence. While the analyses do not provide information about what other characteristics are associated with not transitioning to lower- risk profiles, this would be a fruitful area for future research. In particular, identifying individuals unlikely to respond to a relatively brief ME indicated prevention program may provide a basis for prescreening and funneling these individuals to alternative interventions that may be more successful. For example, these may be individuals whose substance use is such that they might benefit from more intensive individual treatment. Conversely, not all heavy drinking people may need additional treatment. For some, indicated prevention appears to be enough.

### Study limitations

Readers should consider this study’s results in the context of its limitations. First, the data were self-reported and did not include corroboration by other means (e.g., biomarkers or collateral reports). Second, they solely reflect future intentions at postintervention, and we do not know about longer-term outcomes, including behavior. Given that intentions are known to be correlated with subsequent behavior only to a moderate extent, it is likely that a portion of individuals who intended to make positive changes later failed to do so. Since behavioral and other outcomes are unknown, future research should apply these techniques using follow-up data on actual drinking behavior. Third, the analyses included no comparison group; therefore, caution should be used in interpreting the benefits shown until replication can occur in an experimental study. Finally, we do not know the extent to which the distribution of questionnaires by instructors, the legal requirement to attend the program, or other unmeasured factors may have influenced the results.

## Conclusions

These analyses provide information about an important population: emerging adults with an impaired driving arrest. Interventions with this group are not only important for public safety, but also have public health value in that some members of this group are likely to have developed—or be developing—longer-term substance use problems. Indeed, some participants did report preintervention problematic drinking profiles despite having experienced the negative consequence of an arrest. The results show that short-term changes can occur in this important—and sometimes hard to influence—age group during the course of ME intervention (in this case, PFL). Additionally, analyses provide clues to preferred methods of risk reduction. Future research can extend this by describing the longer-term outcomes, and can profit from this approach of categorizing individuals according to their drinking profiles.

## Abbreviations

AIC: Akaike information criterion
BIC: Bayesian information criterion
EA: emerging adulthood/emerging adult
HR: high risk
LCA: latent class analysis
LR: low risk
LTA: latent transition analysis
ME: motivation-enhancing
MI: motivational interviewing
OR: odds ratio
OUI: operating a vehicle under the influence of alcohol or drugs
PFL: Prime For Life

## References

[1] Arnett J (2000). Emerging adulthood: a theory of development from the late teens through the twenties. Am Psychol.

[2] Baer J, Peterson P, Miller W, Rollnick S (2002). Motivational interviewing with adolescents and young adults. Motivational interviewing: preparing people for change.

[3] Arnett J (2005). The developmental context of substance use in emerging adulthood. J Drug Issues.

[4] Casswell S, Pledger M, Pratap S (2002). Trajectories of drinking from 18 to 26 years: identification and prediction. Addiction.

[5] Johnston LD, O’Malley PM, Bachman JG, Schulenberg JE. Monitoring the Future National Survey results on drug use, 1975–2003 (NIH Publication No. 04-5508). Bethesda: Department of Health and Human Services. 2003. http://monitoringthefuture.org/pubs/monographs/vol2_2003.pdf. Accessed 3 Oct 2015.

[6] Auerbach KA, Collins LM (2006). A multidimensional developmental model of alcohol use during emerging adulthood. J Stud Alcohol.

[7] Cleveland MJ, Lanza ST, Ray AE, Turrisi R, Mallet KA (2012). Transitions in first-year college student drinking behaviors: does pre-college drinking moderate the effects of parent- and peer-based intervention components?. Psychol Addict Behav.

[8] Cleveland MJ, Mallett KA, White HR, Turrisi R, Favero S (2013). Patterns of alcohol use and related consequences in non-college-attending emerging adults. J Stud Alcohol.

[9] Henson JM, Pearson MR, Carey KB (2015). Defining and characterizing differences in college alcohol intervention efficacy: a growth mixture modeling application. J Consult Clin Psychol.

[10] Schulenberg JE, Magos JL (2002). A developmental perspective on alcohol use and heavy drinking during adolescence and the transition to young adulthood. J Stud Alcohol.

[11] Flay B, Biglan A, Boruch R, Castro F, Gottfredson D, Kellam S (2005). Standards of evidence: criteria for efficacy, effectiveness, and dissemination. Prev Sci.

[12] Jacobson N, Truax P (1991). Clinical significance: a statistical approach to defining meaningful change in psychotherapy research. J Consult Clin Psychol.

[13] Miller W, Rollnick S (2013). Motivational interviewing: helping people change.

[14] Cronce J, Larimer M (2011). Individual-focused approaches to the prevention of college student drinking. Alcohol Res Health.

[15] Hustad J, Mastroleo N, Kong L, Urwin R, Zeman S, LaSalle L (2014). The comparative effectiveness of individual and group brief motivational interventions for mandated college students. Psychol Addict Behav.

[16] Larimer M, Cronce J (2002). Identification, prevention, and treatment: a review of individual-focused strategies to reduce problematic alcohol consumption by college students. J Stud Alcohol Suppl.

[17] Larimer M, Cronce J (2007). Identification, prevention, and treatment revisited: individual-focused college drinking prevention strategies 1999–2006. Addict Behav.

[18] Tanner-Smith E, Lipsey M (2015). Brief alcohol interventions for adolescents and young adults: a systematic review and meta-analysis. J Subst Abuse Treat.

[19] Prochaska J, DiClemente C (1983). Stages and processes of self-change of smoking: toward an integrative model of change. J Consult Clin Psychol.

[20] Sheeran P (2002). Intention-behavior relations: a conceptual and empirical review. Eur Rev Soc Psychol.

[21] Fishbein M, Ajzen I (1975). Belief, attitude, intention, and behavior: an introduction to theory and research.

[22] Ajzen I (1991). The theory of planned behavior. Organ Behav Hum Decis.

[23] Beadnell B, Nason M, Stafford P, Rosengren D, Daugherty R (2012). Short-term outcomes of a motivation-enhancing approach to DUI intervention. Accid Anal Prev.

[24] Beadnell B, Crisafulli MA, Stafford PA, Rosengren DB, DiClemente CC (2015). Operating under the influence: three year recidivism rates for a motivation-enhancing intervention versus standard care programs. Accid Anal Prev.

[25] Greenfield T, Kerr W (2008). Alcohol measurement methodology in epidemiology: recent advances and opportunities. Addiction.

[26] Greenfield T, Nayak M, Bond J, Ye Y, Midanik L (2006). Maximum quantity consumed and alcohol-related problems: assessing the most alcohol drunk with two measures. Alcohol Clin Exp Res.

[27] Rosengren DB, Beadnell B, Nason M, Stafford P, Daugherty R (2012). Reports of past alcohol and drug use following participation in a motivation enhancing intervention: implications for clinical assessment and program evaluation. Subst Abuse Treat Prev Policy.

[28] Stinchfield R (1997). Measurement, instruments, scales, and tests: reliability of adolescent self-reported pretreatment alcohol and other drug use. Subst Use Misuse.

[29] Del Boca FK, Darkes J (2003). The validity of self-reports of alcohol consumption: state of the science and challenges for research. Addiction.

[30] Daugherty R, O’Bryan T (2004). PRIME for life instructor manual version 8.

[31] Daugherty R, Leukefeld C, Gullotta T, Bloom M (2003). Risk reduction strategies to prevent alcohol and drug problems in adulthood. The encyclopedia of primary prevention and health promotion.

[32] DiClemente C (2003). Addiction and change: how addictions develop and addicted people recover.

[33] Petty R, Brinol P (2008). Persuasion: from single to multiple metacognitive processes. Perspect Psychol Sci.

[34] Collins LM, Lanza ST (2010). Latent class and latent transition analysis.

[35] Muthén B, Satorra A (1995). Complex sample data in structural equation modeling. Sociol Methodol.

[36] Velasquez M, Crouch C, Stephens N, DiClemente C (2015). Group treatment for substance use.

